# Experiences with rotavirus vaccines: can we improve rotavirus vaccine impact in developing countries?

**DOI:** 10.1080/21645515.2018.1553593

**Published:** 2019-02-08

**Authors:** A.D. Steele, J.C. Victor, M.E. Carey, J.E. Tate, D.E. Atherly, C. Pecenka, Z. Diaz, U.D. Parashar, C.D. Kirkwood

**Affiliations:** a Enteric and Diarrheal Diseases, Bill & Melinda Gates Foundation, Seattle, WA, USA; b Policy, Access and Innovation, Center for Vaccine Innovation and Access, Seattle, WA, USA; c Division of Viral Diseases, Centers for Disease Control and Prevention, Atlanta, GA, USA

**Keywords:** Rotavirus vaccines, vaccine impact, cost-effectiveness, Rotarix™, RotaTeq™, RotaVac™

## Abstract

Rotavirus vaccines have been introduced into over 95 countries globally and demonstrate substantial impact in reducing diarrheal mortality and diarrheal hospitalizations in young children. The vaccines are also considered by WHO as “very cost effective” interventions for young children, particularly in countries with high diarrheal disease burden. Yet the full potential impact of rotavirus immunization is yet to be realized. Large countries with big birth cohorts and where disease burden is high in Africa and Asia have not yet implemented rotavirus vaccines at all or at scale. Significant advances have been made demonstrating the impact of the vaccines in low- and lower-middle income countries, yet the modest effectiveness of the vaccines in these settings is challenging. Current research highlights these challenges and considers alternative strategies to overcome them, including alternative immunization schedules and host factors that may inform us of new opportunities.

## Introduction

There have been significant reductions in global all-cause mortality in young children under five years of age (Under 5) over the last three decades;^1^ yet a common childhood illness – diarrhea – remains a leading infectious cause of pediatric death in children between 1 month and 5 years of age.^2^ Rotavirus is the leading cause of childhood diarrhea and is associated with significant mortality from severe gastroenteritis and rapid dehydration, with the latest annual rotavirus mortality estimates ranging from ~122,322 to 215,757.^3–5^ Analysis of the differences driving these different ranges by the three groups (i.e. Global Burden of Disease (GBD) at the Institute of Health Metrics and Evaluation, University of Washington; Maternal and Child Epidemiology Estimation (MCEE) of the World Health Organization (WHO); and the WHO and US Centers for Disease Control and Prevention (CDC) estimates), has helped our understanding of what these drivers are and offers a transparent platform on which to build future estimates.^6^ Nevertheless, the highest rates of rotavirus mortality and hospitalization are seen in low-income countries (LIC), particularly in sub-Saharan Africa and South Asia, and five countries account for >50% of all rotavirus deaths (India, Nigeria, Pakistan, Democratic Republic of Congo and Angola).^4^

A global analysis of the public health impact of rotavirus vaccines indicated that vaccination in the 72 Gavi-eligible countries would prevent 2.4 million childhood deaths and avert 83 million disability-adjusted life years (DALYs) between 2011 and 2030.^6^ More than 95% of the averted burden was determined to be in the African, Eastern Mediterranean and South Asian regions. Furthermore, immunization was considered a “very cost effective” intervention for the entire Gavi birth cohort at $42/DALY averted, although differences were noted in different regions. That finding was based upon the then commonly used international definitions for a “very cost effective” intervention (i.e. when the cost per DALY averted is less than the GDP per capita of that country) to demonstrate that rotavirus vaccine was a very cost-effective intervention for every region.^7^ Recent analysis has encouraged countries to utilize these cost-effective ratios within country-specific context and data to inform processes for decision-making.^8^

Two more recent modeled estimates of the potential impact of rotavirus vaccine in Africa^9^ and Asia,^10^ used current data on regional rotavirus mortality, vaccine effectiveness in introducing countries and observed vaccine coverage. In the 29 African countries that had introduced rotavirus vaccine, an estimated 21,000 deaths and 135,000 hospitalizations were prevented in 2016.^9^ However, if all African countries, including the Democratic Republic of Congo (DRC) and Nigeria had introduced vaccine by December 2014, then an *additional* 139,000 rotavirus hospitalizations and approximately 27,000 deaths would also have been prevented in 2016. Similar potential incremental impact was estimated for countries in the Asian region where rotavirus vaccines could have prevented over 700,000 hospitalizations and 35,000 deaths due to rotavirus in 2016, if the vaccine had been introduced in all countries before the end of 2014.^10^ Notably, India and Pakistan have introduced rotavirus vaccines in a phased manner nationally and Bangladesh is yet to introduce, although it has been approved for support by Gavi.

Two internationally licensed and globally available rotavirus vaccines have been pre-qualified by the World Health Organization (WHO), are licensed in >100 countries, and are being introduced into the routine Expanded Program for Immunization (EPI) schedules of countries. Rotarix™ (GSK Biologicals, Rixensart) is a monovalent (G1P8) human rotavirus strain, which has demonstrated high efficacy and acceptable safety in clinical trials in Europe, Latin America, high-income countries in Asia and Africa using a novel 2-dose schedule for infants.^11–14^ RotaTeq™ (Merck & Co., Whitehouse, Pennsylvania) is a pentavalent bovine-human reassortant rotavirus vaccine, carrying neutralization epitopes against the common human rotavirus genotypes (G1–G4 and P8 ^7^). It has also demonstrated high efficacy and acceptable safety in clinical trials in the US, Europe, Africa and Asia using the traditional 3-dose EPI schedule for infants.^15–17^ Based on the high burden of disease in developing countries and on the efficacy of the vaccines to confer protection against moderate to severe rotavirus diarrhea, in 2009 WHO recommended the introduction of rotavirus vaccines in all countries and particularly in countries with high diarrheal disease mortality.^18^ A third rotavirus vaccine, ROTAVAC™ (Bharat Biotech, Hyderabad, India) obtained WHO prequalification in January 2018, based on a safety and efficacy trial conducted in India.^19^ India commenced rotavirus vaccine introduction in a phased national introduction in 9 states commencing in 2016, where rotavirus vaccine has been provided as part of the routine immunization program. A second locally produced and licensed rotavirus vaccine (RotaSIIL, Serum Institute of India, Pune) was introduced into a 10^th^ state in early 2018. RotaSIIL was WHO prequalified in September 2018.

## Progress with rotavirus vaccines

**Figure 1.**
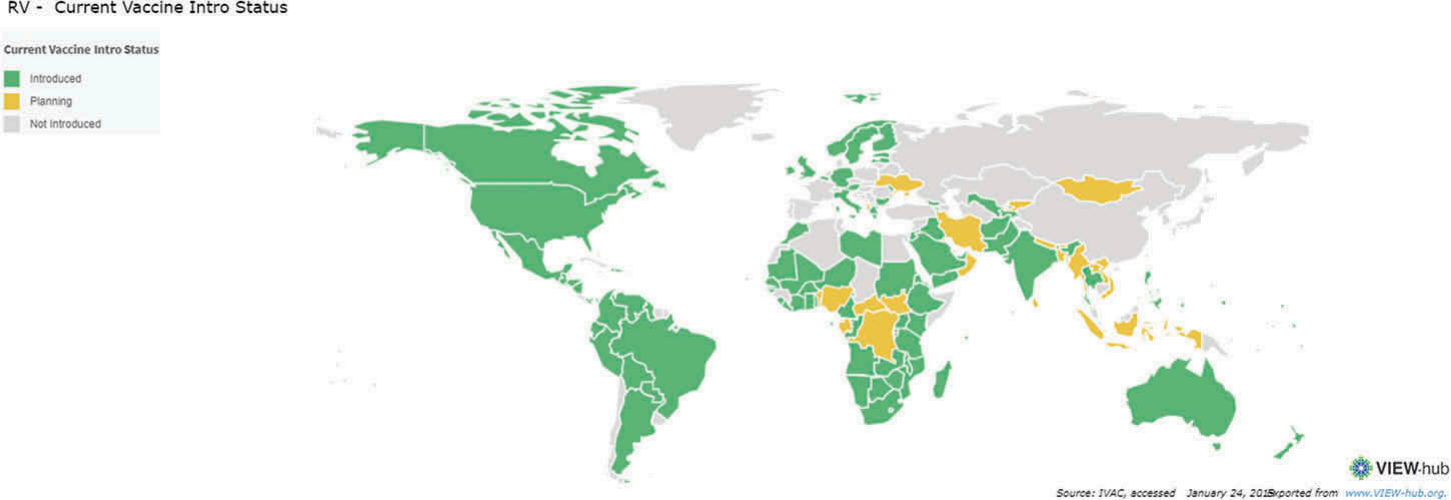
Global introduction of rotavirus vaccines. http://view-hub.org/viz/#

Global introduction of rotavirus vaccination programs and demonstration of their impact have been among the most rapid in history. Currently, 95 countries have introduced rotavirus vaccine into their national childhood immunization programs, including 45 Gavi-eligible countries which utilized financing support from the Gavi Alliance for vaccine procurement (Figure 1). An additional five countries (Canada, Sweden, Italy, Philippines and Thailand) have used the vaccines regionally. Reassuringly, rotavirus disease and hospitalizations have been consistently reduced in countries that have introduced the vaccines in both high-income (HIC) and upper middle-income countries (UMIC).^20–22^ Furthermore, country analyses have shown real reductions in diarrhea-related mortality over time, such as in Mexico (35%) and Brazil (22%) after rotavirus vaccine introduction,^23,24^ demonstrating the powerful impact of rotavirus immunization.

However, the 45 Gavi supported introductions constitute only ~35% of the Gavi birth cohort, and while uptake in sub-Saharan Africa has been widespread, there has been a notable lack of implementation in countries with the highest burden in South Asia, (e.g. Afghanistan only introduced in 2017, India and Pakistan have introduced in a phased approach, and Bangladesh which is yet to introduce despite approval for support by Gavi). Furthermore, in countries with the highest rates of rotavirus-associated mortality in Africa – such as Nigeria and Democratic Republic of Congo (DRC), rotavirus vaccine implementation has not occurred.

In 2016, India introduced their indigenously developed and manufactured rotavirus vaccine, ROTAVAC™,^25^ into four early-adopter states to assess the programmatic feasibility of adding a new oral vaccine to their Universal Immunization Program (UIP). In 2017 and 2018, additional states have been included and nation-wide roll out is anticipated in 2019 which will include a second Indian-licensed rotavirus vaccine from Serum Institute. Similarly, several other countries with large birth cohorts such as China, Indonesia and Vietnam also have their own domestic rotavirus vaccine development programs and have not yet introduced vaccine nationally.^26^

There are several reasons why countries may not have adopted rotavirus immunization. These reasons are multifactorial but include the modest efficacy of the vaccines (45–65% in countries with high Under 5 childhood mortality);^27^ vaccine pricing and financing concerns; programmatic readiness (e.g. insufficient cold-chain capacity, etc); the lack of a country-specific cost effectiveness data, and global supply considerations. To achieve the full potential global impact that could be expected from rotavirus vaccines, we need to consider each of these issues.

## Challenges with the globally available rotavirus vaccines

Twelve years after the first introductions of rotavirus vaccines in 2006, questions remain including: the effectiveness of these live, attenuated oral vaccines in impoverished populations with little access to medical care and high disease co-morbidities including low income and lower-middle income countries (LIC; LMIC), and impoverished communities in some relatively wealthy countries; the duration of protection beyond the first year of life; the possibility of indirect benefits through herd protection of unvaccinated children in high burden settings; and the cross protection against the dynamically evolving variety of rotavirus strains (particularly for the human monovalent vaccine). Two additional questions relating to the young infant have also been raised: the role of potential interference of maternal antibody either transplacentally acquired or via breastfeeding on the effectiveness of the vaccines; and whether environmental enteropathy of the infant gut restricts the live vaccine responses. Specific studies have been conducted over the past 3–5 years to directly address each of these questions.

### Vaccine effectiveness in lower-middle and low-income settings

Despite modest clinical efficacy observed in randomized, controlled trials in developing countries in Africa and Asia, ranging from approximately 45–65%,^14,16,17,28^ recent studies have documented the real-world impact of the vaccines once introduced into the routine immunization schedule of LICs and LMICs (Table 1).^29^ For instance, several Gavi-eligible countries in Latin America including Bolivia, Honduras and Nicaragua demonstrated reductions in diarrhea-related mortality after rotavirus vaccine introduction, compared to no overall reductions in countries that did not introduce the vaccine (Argentina, Chile, Costa Rico, Paraguay).^30^ These declining trends in diarrhea mortality post rotavirus vaccine introduction, were notable in infants (<12 months of age) ranging from 30–48% in Nicaragua and El Salvador respectively; and were also observed in children <5 years of age (36–50%) in Nicaragua and El Salvador in the 4-year period post rotavirus vaccine introduction.^30^

Moreover, dramatic reductions in rotavirus-associated hospitalizations and in all-cause diarrheal hospitalizations have been documented in several LMICs in Latin America, central Europe and Africa.^31–38^ The first LICs to document the effectiveness of rotavirus vaccines have also demonstrated the successful public health impact of the vaccines. For instance, in Malawi, which introduced the monovalent Rotarix™ vaccine in 2012, rotavirus-related hospital admissions decreased by >40%.^37^ Rwanda introduced the pentavalent RotaTeq™ vaccine in 2012, showing similar reductions for acute diarrhea hospital admissions (48–49%) and rotavirus-specific admissions (61–70%).^38^ These observations of the significant impact of rotavirus vaccines should encourage countries with high diarrheal burden to introduce the vaccines as a priority.

Finally, the cost effectiveness of the vaccines has been favorably evaluated in numerous settings including LIC and LMIC countries.^39,40^ In Bolivia for instance, a cost effectiveness analysis was performed to support country decision making in 2017, when it is likely that vaccine subsidy support may be reconsidered. Rotavirus vaccine was demonstrated to be cost effective in Bolivia at their current price in 2011 (US$9/dose) and cost saving at US$3.81 per dose.^41^ It was noted that rotavirus vaccination at the then current price of US$9 per dose was comparatively more cost-effective than most other early childhood interventions including expansion of oral rehydration therapy or vitamin A supplementation.^41^

In several African countries, cost effectiveness analyses have also demonstrated the benefit of rotavirus vaccination at both the State and the family level. Ngabo et al.^42^ calculated the economic burden of a hospital admission for diarrhea in Rwanda to be US$101, of which direct medical costs were US$44.22 (±US$23.74), and approximately two-thirds of costs (65%) was borne by the household. For the lowest income group, these costs exceeded the household’s monthly income. Several other analyses demonstrated that this is not unique to Rwanda.^43,44^ The cost per disability-adjusted life year (DALY) was documented to be very cost effective (defined as costing less than the Gross Domestic Product (GDP) per DALY averted) or cost effective (<3x the GDP per DALY averted) in several countries in Africa including Senegal, Kenya, Uganda, Malawi and Ghana.^43–46^ In Ghana, a recent analysis documented that rotavirus immunization will remain very cost effective for the country even after Gavi support ends.^46^ In addition, several recent analyses in Asia have documented that rotavirus vaccination would be highly cost effective in Afghanistan, Bangladesh, Laos and Pakistan.^47–50^ In Afghanistan, a high mortality setting, rotavirus immunization with or without Gavi support is very cost effective, representing <3% of the country’s immunization budget. Furthermore, the costs per DALY averted are approximately $30/DALY averted when utilizing the Gavi subsidy.^47^ In Bangladesh, representing a low diarrhea mortality setting, rotavirus vaccines were also cost effective with or without Gavi subsidy support.^48^ Finally, Rheingans and colleagues report that the benefits and cost effectiveness of a rotavirus vaccine program in Pakistan, can be maximized by reaching the highest risk population groups.^50^

### Duration of protection beyond the first year of life

Several lines of evidence suggest that vaccine-acquired protection is possibly not as enduring or complete as initially predicted. In the African clinical studies, for instance, vaccine efficacy was lower in the second year of life in all settings,^16,51,52^ although this was not observed in two Asian studies.^17,53^ Recent post-hoc analyses of the placebo groups in vaccine trials conducted in Africa, Asia and Europe may shed some light on these observations. Early exposure to wildtype rotavirus infection was demonstrated in a review of the placebo groups within clinical studies, indicating that approximately 25% of infants in Malawi and South Africa had been exposed to natural infection by 20–24 weeks of age.^54^ This was similar in India although Bangladeshi infants showed the highest rates of early exposure by 20 weeks of age (35%). However, at approximately 6–8 weeks of age, the Asian infants had higher rates of exposure (18–25%) compared to African infants (11–13%), Latin American infants (2–5%) and European infants (0–2%),^54^ shown in Table 2. A separate post-hoc analysis on the incidence of rotavirus gastroenteritis episodes in various settings, showed that the incidence of any and severe rotavirus gastroenteritis (defined by the Vesikari severity score of ≥11^14^) was higher in African infants in the first year of life, than European or HIC Asian infants.^55^ In addition, the first severe rotavirus episode seemed to occur at a younger age in African children. An earlier review demonstrated that >80% of the rotavirus symptomatic disease occurred in African infants before their first birthday.^56^ During the second year of life, the incidence of any or severe rotavirus gastroenteritis was lower in Africa, suggesting that these infants were protected by repeated natural infection. Conversely, approximately a third of rotavirus disease was observed in the second year of life in Asian infants from impoverished settings,^57^ despite similar early exposure. A birth cohort study conducted in Vellore, India, indicated that infants were subject to multiple symptomatic rotavirus episodes in the first 2 years of life, even by the same viral strain.^58^ It is reasonable to think that differences in the force of infection and pre-existing antibody levels, and possibly access to medical care such as oral rehydration, may play a role in these observed differences in exposure and severe infection.

**Table 1.** Vaccine effectiveness against hospitalization or hospitalization and emergency department attendance rotavirus gastroenteritis in low income and low-middle income settings.

			VE (95% CI)	
Country	vaccine	outcome	<12mo	>12mo
Armenia	Rix	hospital	68 (24,86)	60 (20,80)
Bolivia	Rix	hospital	64 (34,80)	72 (52,86)
Bolivia	Rix	hospital	76 (50,89)	47 (0,70)
Botswana	Rix	hospital	52 (8,75)	67 (8,89)
Brazil	Rix	hospital, ER	81 (47,93)	5 (−187,69)
Brazil	Rix	hospital	56 (12,78)	32 (−4,56)
Brazil	Rix	hospital	74 (58,84)	78 (54,90)
Colombia	Rix	hospital	84 (23,97)	−79 (−559,51)
El Salvador	Rix	hospital	83 (68,91)	59 (27,77)
Ghana	Rix	hospital	78 (2,95)	50 (−57,84)
Guatemala	Rix	hospital, ER	74 (18,92)	71 (44,85)
Malawi	Rix	hospital	71 (34,87)	32 (−141,81)
Malawi	Rix	hospital	62 (28,80)	31 (−139,80)
Moldova	Rix	hospital	84 (67,92)	46 (−16,75)
South Africa	Rix	hospital	54 (32,68)	61 (35,77)
Tanzania	Rix	hospital	56 (−2,81)	57 (−30,86)
Burkino Faso	Teq	hospital	58 (10,81)	19 (−78,63)
Guatamala	Teq	hospital, ER	74 (18,92)	71 (44,85)
Nicaragua	Teq	hospital, ER	78 (49,91)	55 (22,74)
Nicaragua	Teq	hospital	64 (43,78)	30 (−5,53)
Nicaragua	Teq	hospital, OP	65 (−80,93)	81 (25,95)

Footnote: adapted from reference 30

**Table 2.** Age of exposure to rotavirus infection in young children globally, as assessed by exposure in the placebo groups of vaccine studies.

		Sero-positive status	Sero-positive status
Region	Country	at 6–8 weeks of age^1^	at 20–24 weeks of age
Africa	South Africa/Malawi	11%	17%
	Ghana/Kenya/Mali	2%	20%
Asia	Bangladesh	15%	35%
	India	26%	26%
Latin	Brazil/Mexico/Venezuela	2%	13%
America	Multiple countries^3^	4%	15%
Europe	Finland	0%	0%
	Multiple countries	2%	9%
United States		0%	9%

^1^Sero-positivity assessed by to anti-rotavirus IgA antibody titres

^2^Blood was drawn 4– 8 weeks post last vaccine administration

^3^Argentina, Brazil, Chile, Colombia, Dominica Republic, Honduras, Mexico, Nicaragua, Panama, Peru, Venezuela

^4^Czech Republic, Finland, France, Germany, Italy, Spain

Footnote: adapted from references 55 and 56

Similarly, in effectiveness studies conducted after introduction, differences were noted in some settings but not in others. For instance, in Nicaragua, protection waned in the second year of life^31,59^ compared to the US where it did not.^60^ In Bolivia, a case control study showed that vaccine effectiveness was diminished in children >12 months of age compared to those <12 months of age, although this trend was not observed for *very* severe rotavirus diarrhea.^61^ In Guatemala, a case control study using hospital controls, reported no significant difference in effectiveness between infants 6–12 months of age and children >12 months.^62^

Several African countries have reported similar observations in the 2–3 years’ post introduction, supporting higher impact of the vaccine on hospitalizations in the first year of life, and generally the impact was highest against the more severe disease. Interestingly, vaccine effectiveness in South Africa, where Rotarix™ was administered at an alternative schedule of 6 and 14 weeks of age, was demonstrated to have significant impact against rotavirus gastroenteritis and all-cause diarrhea hospital admissions in both the first year of life and persisted through the second year of life^63^ and was documented in both HIV-infected and HIV-uninfected infants.^64^ Thus, these combined observations suggest that the vaccine, administered at a longer interval between doses, was protective against the most severe rotavirus disease in the first two years of life, which is arguably the most significant public health issue. Certainly, increasing evidence in multiple settings demonstrates that the vaccines prevent rotavirus-associated mortality and hospitalizations.^65^

Intriguingly, a decrease in vaccine effectiveness was observed during rotavirus outbreaks in 2009 and 2010 in the Northern Territories of Australia. This low vaccine effectiveness was associated with the emergence of rotavirus antigenic variants, providing the first evidence of rotavirus vaccine escape mutants.^66^ This suggests that there are multiple scenarios related to the duration of protection afforded by rotavirus vaccines, including possible modest protection in infants in high burden settings, or the emergence of vaccine escape strains, which remains to be quantified as a threat.

### Indirect protection in unvaccinated persons

Early indications of indirect protection of unvaccinated children was observed in the US within two years of vaccine introduction,^67,68^ where reductions in rotavirus gastroenteritis were observed in children who were ineligible for vaccination due to their age (i.e. greater than 6 months of age), and in a greater proportion of children <5 years than had been initially been vaccinated. Indirect protection of almost 50% was also noted in US adults who were clearly not vaccinated, demonstrating a broader public health impact.^69^ This indirect protection has been documented in other HIC settings in Australia and Europe,^70–72^ highlighting the potential added benefit of rotavirus immunization. Furthermore, in South Africa, a vaccine impact study conducted after the introduction of Rotarix™ in 2009, demonstrated reductions in children between 12 and 24 months of age, who were too old to be vaccinated.^73^

The remaining question is whether significant indirect protection will be observed in LMICs and LICs where the force of infection is far higher. A cluster randomized trial of Rotarix introduction in rural Bangladesh attempted to measure indirect protection but found none.^74^ The authors noted that larger scale sustained vaccination may have been required in such a high force of infection setting to obtain measurable indirect effects. Early data from country introductions in Armenia and Moldova suggest that indirect effects may be observed in some settings in LMICs.^32,33^ It is likely that the level of indirect protection will depend on the intensity of rotavirus transmission in a population, coverage with vaccine, and the level of protection afforded to vaccinees.

### Cross protection of dynamically evolving rotavirus strains

Rotavirus ‘genotypes’ are defined by gene sequences of the two outer capsid proteins; namely the VP7 glycoprotein outer capsid protein (G-types), and the VP4 protease-sensitive viral receptor which protrudes as a spike from the surface of the particle (P-types). The genotype characteristics of the VP7 (G for glycoprotein) and VP4 (P for protease-sensitive) are identified by reverse transcriptase PCR of portions of the genes encoding these two major external antigens of the viral particle which are considered important neutralization antigens.^75^

In the pre-vaccine era, rotavirus strains underwent temporal and spatial changes each year within and between countries. In the past two decades, six rotavirus genotypes were identified as the common human virus strains causing disease – G1P[8], G2P[4], G3P[8], G4P[8], G9P[8] and G12P[8].^75,76^ Genotype G1P[8] strains are consistently present and predominate globally, representing ~70% of strains in developed countries, but only 25–50% in countries in South America, Africa and the Indian sub-continent.^76–78^

Both vaccines have demonstrated effective clinical protection against multiple homotypic or heterotypic rotavirus strains. For the monovalent (G1P[8]) Rotarix™ vaccine, heterotypic cross protection was a specific concern, but the vaccine was protective against completely heterologous strains – G2P[4], G8P[6] and G12P[6] in developing countries.^79,80^ Similarly, the pentavalent RotaTeq™ vaccine (G1-G4 and P[8]) has shown protection against non-vaccine strains.^15,81^ Nevertheless, there has been much conjecture whether widespread use of rotavirus vaccines would result in evolutionary selective pressure resulting in strain replacement, as was seen after the introduction of pneumococcal vaccine. Early observations of the strains circulating in countries after rotavirus vaccine introduction hint at the potential for strain replacement. Findings in Australia, Belgium, Latin America and the USA have revealed significant changes in the diversity and distribution of the circulating wildtype rotavirus population following vaccine introduction with both Rotarix™ or RotaTeq™.^82^

For example, in the post-vaccine era genotype G2P[4] strains emerged as a common cause of disease in several settings, and circulated in a prolonged manner over several years,^83^ compared to earlier observations that G2P[4] strains tended to occur in a cyclic manner every 2–3 years. Similarly, G3P[8] strains emerged as a more common genotype in the post-vaccine era, in contrast to its relatively minor role in the pre-vaccine era.^84,85^ Furthermore, genotyping data post vaccine introduction revealed the identification of previously unrecognized genotype recombinants, such as G3P[14].^86,87^ A recent detailed phylogenetic analysis of the full genome analysis of G1P[8] strains from Belgium and Australia post-introduction showed evidence of specific, distinct viral sub-clusters present before and after vaccine introduction. Interestingly, the emergence of unique viral clusters identified only after vaccine introduction may be the first sign of vaccine-induced evolutionary pressure.^88^

Thus, molecular epidemiological and bioinformatics studies indicate that rotavirus genotypes appear to be more diverse and dynamic following vaccine introduction. The shift in strain diversity may be due to natural annual fluctuations of the circulating strains which has been widely documented,^75–78^ although the distinct distribution patterns and divergent strains emerging in the vaccine era appear unique and suggest that mass vaccination has had an impact on circulating genotype diversity.

However, this complex question requires careful consideration of three important issues for the global public health impact of rotavirus vaccination. First, rotavirus vaccination prevents disease, but does not result in sterilising immunity and thus subsequent re-infection occurs. This is similar to the natural history of the disease where children are infected multiple times during the first few years of life, but most of these subsequent infections are sub-clinical and non-life threatening. While it is true that vaccination may generate an immune environment that will exert novel immunological pressures on the circulating wildtype rotavirus strain population and may alter the dynamic nature that drives strain selection, as described above, it is not apparent that this will make current rotavirus vaccines obsolete. Secondly, it is important to note that rotavirus induces immune responses to other viral proteins including the non-structural proteins NSP2 and NSP4,^89,90^ indicating that protection is affected by more than just neutralizing antibody to the two outer capsid proteins. Protective immune responses found after live rotavirus immunization could be stimulated by either B- or T-cell epitopes present on any rotavirus protein, and these epitopes may be conserved within different rotavirus serotypes.^91^

Finally, the question of whether vaccine-driven strain replacement may become a global public health concern, when weighed against the dynamic natural history and distribution of rotavirus genotypes was addressed recently. Mathematical modelling assessed data on genotype-specific hospitalizations for rotavirus diarrhea in Belgium to examine the underlying dynamics driving changes in the genotype distributions before and after vaccine introduction.^92^ Rotavirus vaccines were introduced in the national immunization schedule of Belgium in 2006 and vaccine coverage, primarily with the monovalent Rotarix™ vaccine, was high (>85%), resulting in a dramatic drop in hospitalization incidence for rotavirus gastroenteritis. The model estimated that natural- and vaccine-derived immunity was strongest against completely homotypic G1P[8] strains and weakest against completely heterotypic G2P[4] strains. The predominance of G2P[4] infections after vaccine introduction was explained by a combination of natural genotype fluctuations and slightly weaker natural- and vaccine-induced immunity against strains heterotypic to the vaccine. However, the incidence of rotavirus gastroenteritis declined dramatically and is predicted to remain low despite possible changes in the relative distribution of genotypes.^92^ Current reductions in rotavirus hospitalizations in multiple LIC and LMIC settings where rotavirus strain diversity is high, confirm that strain replacement, while a potential threat to the current rotavirus vaccines may not occur, but requires continued monitoring to ensure that we see the continued impact of the current or new rotavirus vaccines.

### Vaccine interference by maternal antibody

Several studies have evaluated the role of maternal antibody, whether transplacentally-acquired or via breastfeeding, on the response to rotavirus vaccines. Initial reports of the impact of maternal antibody on the replication of live attenuated rotavirus vaccines *in vitro* fueled a flurry of studies evaluating this specific question. First, several studies have shown that maternal IgG titres of anti-rotavirus antibody is important early in life and is reflected in passively acquired infant IgG titres. This transplacentally-acquired antibody protects the very young infant; and the maternal serum IgG titres are negatively correlated with the immune response of the infant to the vaccines.^93–96^ High levels of pre-existing serum IgG in the infant, including maternally derived IgG, have an inhibitory effect on the immunogenicity of both the monovalent human rotavirus vaccine,^96^ and the pentavalent reassortant vaccine.^95^

In addition, several studies have explored the role of maternal antibody acquired through breastfeeding – both as an active inhibitor of viral replication at the time of administration of the oral, live vaccine, and assessing the impact of high titres of anti-rotavirus breastmilk antibody on vaccine take.^97–99^ Anti-rotavirus IgA and IgG are generally higher in women in impoverished settings, presumably due to constant exposure to wildtype rotavirus and have been recorded to negatively impact the replication of the vaccine in the infant gut.^93,96,97^ However, studies assessing the effect of withholding breastfeeding before and after actual vaccine administration did not show a negative impact of the concomitant breastfeeding,^97–100^ and in fact one study showed slightly better IgA immune responses in those infants fed at the time of administration.^100^ Thus, although breastmilk antibody titres might be higher in some populations and this may have some effect on the first rotavirus vaccine dose, the available data indicates that after the full course of vaccines, this effect appears nullified. The overall benefits of breastfeeding, particularly in young infants in developing countries, far outweigh the limited interference of breastmilk antibody on rotavirus vaccine take. Furthermore, WHO recommends exclusive breastfeeding up until 6 months of age – so over the period that infants receive their childhood immunizations – and recommends continued breastfeeding during a diarrheal episode.

### Environmental enteric of the infant gut

Environmental enteric dysfunction (EED) is a subclinical enteric condition of children in developing countries that is characterized by intestinal inflammation, reduced intestinal absorption and gut barrier dysfunction.^101^ A clinical study of Rotarix™ and oral polio vaccine (OPV) conducted in Bangladeshi infants demonstrated that >80% of the children had signs of EED by 12 weeks of age, based on a range of fecal biomarkers for intestinal inflammation. EED was also associated with malnutrition at 12 months of age (28% of infants had HAZ scores of ≤2), and with micronutrient deficiencies, including zinc. Interestingly, EED was associated with failure to respond to OPV or Rotarix™ vaccination (20% and 69%, respectively), although immune responses to the parenteral vaccines were not impaired.^102^

### Rotavirus, histo-blood group antigens and microbiome

New avenues of research to elucidate the lower efficacy of rotavirus vaccines in developing country infants include an examination of host factors. Recent studies have demonstrated that rotaviruses, like other enteric pathogens, recognize and bind to human histo-blood group antigens (HBGAs) in a strain-dependent manner.^103^ For rotaviruses, the HBGA-binding is mediated by the VP4 outer membrane protein via a glycan-binding site found on the VP8* (a cleavage product of VP4). A recent study exploring these phenomena, examined rotavirus infection in infants from Burkina Faso and Nicaragua, reporting that rotavirus strains with P8 genotypes exclusively infected individuals with Lewis-positive and secretor-positive phenotypes.^104^ Conversely, P6 rotavirus strains predominantly infected Lewis-negative individuals, irrespective of the secretor status. These are intriguing findings because (i) this human phenotype (Lewis-negative) is much more common in African populations, and (ii) the rotavirus P6 genotypes circulate at very high levels in Africa,^76,77^ possibly contributing to the lower efficacy of vaccines carrying the VP4 .^7^ Recently, Lee and colleagues^105^ reported that non-secretor status was associated with reduced risk of rotavirus diarrhea in Bangladesh infants, however, there was no evidence non-secretors were resistant to P8 infection. In this study, reduced VE was mediated by complete protection from P4 infection rather than reduced P8 susceptibility. Further research is needed to understand how significant this issue is.

Harris and colleagues^106^ examined the role of the microbiome in responsiveness to the orally administered vaccine. First, vaccine responders in a clinical study in Ghana,^107^ were matched to non-responders in the same study, using several parameters including age, village, month of vaccination, height and weight. Intriguingly, in this small study, bacteria related to *Streptococcus bovis* were significantly associated with vaccine immune response, and it was speculated that the bacterial family may have acted as an inadvertent adjuvant due to toxigenic lipo-polysaccharide (LPS) and ability to trigger an immune response.^106^ In addition, the Ghanaian microbiome in vaccine non-responders demonstrated higher abundance of bacteria belonging to the *Bacteroidetes* phylum, which includes bacterial species expressing functionally and structurally distinct LPS. This was confirmed in a control group of Dutch infants who were assumed to be high-responders to the vaccine. The signature intestinal microbiota were shared in a small pilot study in Pakistani infants,^108^ but not in Indian infants.^109^ The results are preliminary but indicate that there may be some answers to the lower “vaccine take” submerged in the microbiome of infants in developing countries.

## Can we improve the performance of live oral rotavirus vaccines?

Rotavirus vaccines are based on the concept that live, attenuated vaccine strains that are orally delivered will mimic natural infection resulting in viral replication in the gut and the stimulation of mucosal immunity. Although this principle worked well in developed country infants, we know that these live, attenuated oral vaccines are less effective in developing countries as described above. Several studies have been undertaken to evaluate whether it is possible to enhance the performance of these rotavirus vaccines in developing country infants through changing the immunization schedule or giving additional doses and/or micro-supplementation.

### Immunization schedules with additional doses

Rotarix™ is marketed as a 2-dose product to be administered at 6 and 10 weeks of age. It was initially believed that three doses of a live, oral rotavirus vaccine would be required, and given the existing 3-dose EPI schedule for DPT (now pentavalent vaccine containing DPT-Hib and Hepatitis B vaccine), all rotavirus vaccines were evaluated on a 3-dose regimen, including Rotarix™. However, early studies for the licensure of the Rotarix™ vaccine conducted in Brazil, Mexico and Venezuela and in Finland demonstrated that vaccine gave good protection against severe rotavirus gastroenteritis after 2 doses,^110,111^ and this schedule was utilized in the large pivotal licensure studies.^11–13^

The clinical studies evaluating efficacy and immunogenicity of Rotarix™ in African infants in Malawi and South Africa, were designed with a 2-dose schedule (10 and 14 weeks of age) and a 3-dose schedule (6, 10 and 14 weeks of age) as it was unknown whether the 2-dose schedule would provide sufficient protection in these settings.^14^ The IgA seroconversion rates and geometric mean concentrations (GMCs) were moderately higher in the 3-dose arms, although not statistically significantly so. Vaccine efficacy was higher in the 3-dose arm of the South African cohort, although in Malawi, protection in the first year of life was similar for the two schedules.^14^ However, in the second year of life, protection was significantly higher for infants who received three doses of the vaccine in both countries,^51,52^ suggesting that a 3-dose schedule might have greater public health impact. Nevertheless, the 2-dose schedule was adopted as recommendation policy in 2009 when SAGE reviewed this data, although they did request additional data on this specific question.^18^

Thus, to further investigate the potential benefit of providing three Rotarix™ doses in the primary series, two similarly designed trials were conducted in Pakistan and Ghana using immunologic outcomes. IgA is not a mechanistic correlate of protection for rotavirus but has been routinely used as a surrogate marker to indicate “vaccine take” in all clinical studies. Anti-rotavirus IgA was measured among children receiving Rotarix™ at 6, 10 and 14 weeks of age, compared to that among children receiving vaccine at the recommended 6 and 10 weeks of age, or at the delayed schedule of 10 and 14 weeks of age. In Pakistan, there was no difference between the proportion of infants who seroconverted (3-dose arm, 36.7% compared to 2-dose arm, 36.1%), nor the geometric mean concentrations after the full series in the 3-dose arm (25.8 U/ml) compared to the 2-dose arm (24.0 U/ml).^112^ In contrast, in Ghana, significantly more infants seroconverted in the 3-dose arm compared to the 2-dose arm (43.4% versus 28.9%, p = 0.014). This improvement in immune response was also demonstrated by the GMCs which were significant higher in the 3-dose arm (32.6 U/ml versus 22.1 U/ml, p-0.038).^107^

It is unclear why a third dose of Rotarix™ administered in the primary series stimulated higher immune responses in Ghana, although this is consistent with other studies in African populations studied.^14,51,52,113^ An additional dose of rotavirus vaccine in the primary series may simply provide some children another opportunity to respond to vaccine or could give a modest boost in immunity after initial presentation from the earlier doses. As noted above, there are challenges to live oral vaccines in these infant populations. Given the sub-optimal efficacy that current rotavirus vaccines offer infants and young children in the developing world, even a modest improvement in immunity may translate into substantially more infants protected.

### Booster dose of rotavirus vaccine at 9 months

Another strategy to help overcome interference from maternally-derived antibody and waning immunity in the second year of life, may be the provision of an additional dose of vaccine later in infancy. Investigators in Bangladesh studied the immunologic effect of administration of a booster dose of rotavirus vaccine at 9 months of age with measles/rubella vaccines, to infants who had received two doses of Rotarix™ at 6 and 10 weeks of age.^114^ Importantly, the immune response to measles vaccine was not negatively impacted. Serum IgA and IgG to rotavirus were measured prior to, and two months post the booster dose, demonstrating enhanced rotavirus antibody titres. Infants with negative baseline serum IgA or IgG titres (<20 U/ml), developed seropositive levels ≥20 U/ml after the booster dose, indicating a response to the vaccine in 43.6% (95% CI, 34.7% to 53.0%) and 68.8% (95% CI, 57.9% to 77.9%) of infants, respectively. No changes in serum antibody levels were noted among the group of infants not receiving the booster dose. Most infants demonstrated improvements in anti-rotavirus serum antibody titres, and this was specifically observed among the infants with lowest antibody levels pre-vaccination, and who would likely benefit most, in terms of improved protection, from receipt of the booster dose.^114^ Similar results were observed in Mali.^115^ A study modelling the booster dose strategy has shown that a significant number of additional deaths could be averted if a booster dose re-established VE to the levels seen in year 1 during the second year of life.^116^

### Neonatal dosing for rotavirus vaccines

A novel strategy to avoid interference from maternal antibodies or EED, is to employ a neonatal immunization strategy. A birth vaccine dose may also enhance the coverage and timeliness of vaccine completion, particularly in regions with vaccine programmatic challenges,^117^ although limited evaluation of rotavirus vaccines has occurred in this period. Most recently, the asymptomatic neonatal rotavirus vaccine (RV3-BB) was evaluated in a Phase IIb immunogenicity and efficacy study employing a birth dose (<5 days of age), as part of a 3-dose regime in a randomized double-blinded placebo-controlled study in Indonesia.^118^ Overall, a VE of 63% was observed for 18 months follow-up, when including the infant and neonatal participants together. In the neonatal arm, a VE of 94% was observed at 12 months and 75% at 18 months. These extremely promising efficacy results show the potential of using a neonatal rotavirus strain that was adapted to the newborn gut. Early rotavirus vaccine candidates, which have been discontinued, were evaluated in Finnish and Ghanaian infants showing good immunogenicity and protection against moderate to severe rotavirus gastroenteritis.^119^ Taken together this data suggests that a neonatal administration schedule could be an attractive alternative that could help improve vaccine efficacy and extend protection, particularly given the early exposure to rotavirus infection noted earlier in the placebo groups of trials conducted in developing countries.

### Micro-supplementation with zinc and probiotics

Zinc is critical to immune function, and research demonstrates that zinc deficiency negatively impacts the immune system.^120^ Zinc deficiency is common in low-resource populations in Africa and Asia, and supplementation is proposed as a potential strategy to improve immune responses to vaccinations. Indeed, zinc deficiency was associated with EED and the failure of oral rotavirus vaccine noted above.^102^ Several studies have examined the relationship between zinc and immune responses to childhood immunization, but positive results have been few. In two trials of oral inactivated cholera vaccine in Bangladesh, daily zinc supplementation (20 mg of elemental zinc) for six weeks resulted in a significant increase in the proportion of 2–5 year olds and 10–18-month-old children who had a four-fold or greater increase in vibriocidal antibody titer. However, no effect was measured among infants 6–9 months of age.^121,122^

The intestinal microbiota, as noted earlier, also play a significant role in maturation of gastrointestinal tract health and in mucosal immune function. Lactobacilli are normal commensals of the gut and play a role in regulating the intestinal microbiota and have been regarded as safe and effective probiotics.^123^ Studies in gnotobiotic piglets have demonstrated that *Lactobaillus rhamnosus* GG improves the mucosal barrier function in piglets challenged with rotavirus and enhanced the mucosal B-cell responses to rotavirus challenge.^124,125^ Thus, it has been proposed that the use of probiotics might enhance the mucosal immune responses to live, attenuated rotavirus vaccines that replicate in the gut.

In a clinical trial of zinc supplementation, with or without probiotics, on the response to rotavirus vaccine, infants in India received daily supplementation of elemental zinc from their sixth week of life until 11 weeks of age, and half the infants also received supplementation with a probiotic (10^9^ *Lactobacillus rhamnosus* GG). The study was inconclusive but may have been due to lack of sufficient zinc dosing to show an effect.^126^ It is clear that additional research is required in this area. Most basic research of zinc effects on immune function have centered on the peripheral immune system; there is currently almost no information about the effect of zinc deficiency or supplementation on mucosal immunity.

## Global supply

Currently, there are four WHO prequalified vaccines, which are available for Gavi-eligible countries with financial support from UNICEF for vaccine procurement. In recent years, and based on demonstrated country preference for one product, some short-term supply constraints have been seen.^127^ As several large countries plan to introduce vaccine in 2018 and 2019, this situation is likely to be exacerbated. Based on an earlier assessment by Gavi,^127^ rotavirus vaccine demand by Gavi-eligible countries will increase to 66 million courses per year, and will require the entry of at least one, if not both, additional vaccine manufacturers with prequalified vaccines to meet this demand and that of the non-Gavi-eligible LMIC and UMIC countries. The advent of both Indian pre-qualified vaccines should help this situation, and fortunately, there are several other new vaccine products in development that should offset growing demand for rotavirus vaccine within a few years.

## Conclusions

Live-attenuated, orally administered rotavirus vaccines have repeatedly demonstrated their value in reducing rotavirus-associated deaths and hospitalizations in all settings evaluated. The benefits of rotavirus immunization, particularly in countries with high diarrhea burden, are quantifiable and cannot be denied.^21–38^ Yet, despite these successes, many countries have not introduced rotavirus vaccines.

Rotavirus vaccines have a unique trajectory in that the vaccines were introduced into the United States and Gavi-eligible countries in the same year. In 2006, Nicaragua introduced vaccine with the support of the Pan American Health Organization (PAHO) and the Revolving Fund. The leadership of Nicaragua has continued with multiple assessments indicating the reduction in diarrheal hospitalizations, the cost effectiveness of the vaccine, and the impact of the human monovalent vaccine against heterotypic strains.^31,38,59^ This has paralleled the impact demonstrated in the United States and in other countries in this region over the past 10 years.

Concerns about the modest efficacy of the vaccines in developing countries may have impacted country decision making for vaccine introduction. Several studies over the past 5 years have attempted to understand the role of various host or virus factors that might have played a role in the reduced efficacy observed. In essence, these studies have shown that several factors specific to children in impoverished settings with high exposure do impact the immune response to the vaccines, such as maternal antibody titres,^93–100^ environmental enteric dysfunction,^101,102^ co-administration of oral poliovirus vaccine, etc. Nevertheless, the vaccines still demonstrate good public health impact, and have described additional benefit through indirect herd effects.^64–70^ Furthermore, immune responses can be improved by changes to the number and timing of doses of the vaccine, although enhanced clinical protection has yet to be documented. Effectiveness studies to assess the clinical protection of differing immunization schedules should be considered to enhance the observed benefits. Finally, multiple cost effectiveness studies in various economic settings have demonstrated the clear benefit and cost-effective nature of rotavirus vaccines.^40–50^

Early questions of the cross-protective benefit of the vaccines, particularly the monovalent (G1P[8]) human rotavirus vaccine have also proven unsubstantiated. It is apparent that large scale rotavirus immunization may drive natural evolution and diversity of the viral strains post-immunization,^79–84^ but this has not reduced the overall major effect of the vaccines on reducing diseases and virus transmission.^85,92^ Several analyses have also demonstrated the heterotypic cross-protection of both Rotarix and RotaTeq vaccines against multiple strains and completely heterologous strains. We should continue to monitor the strains circulating post-immunization and assess their potential intrusion as vaccine escape mutants with heightened disease burden.

Finally, countries may delay the decision to introduce rotavirus vaccines, which are relatively costly compared to the older generation of childhood vaccines. Cost effectiveness analyses have established that rotavirus vaccines are very cost-effective (<1 x GDP per DALY-averted) or cost effective (<3x GDP per DALY-averted), to the country, using common criteria for cost-effectiveness.^41–50^ In addition, Gavi subsidy for vaccine procurement through UNICEF, means that the country payment is as low as $0.20 per dose. Non-Gavi LMIC countries have to pay more for vaccine. Cost effectiveness analysis is an extremely powerful tool to assist country-decisions to introduce rotavirus vaccines.

Importantly, countries in Africa have also ramped up introduction of rotavirus vaccines from the first introduction in 2009 in South Africa. The continent houses 9 of the 10 countries with the highest rates of rotavirus mortality per 100,000 population.^4^ Currently, 36 of 54 countries in the continent have introduced rotavirus vaccines into the Expanded Program for Immunization (EPI), and many have documented the major impact of immunization on rotavirus hospitalizations and deaths.^34–37,63,64^ The enormous benefits demonstrated by countries utilizing the vaccines, and the continued support of Gavi and UNICEF for vaccine procurement, will continue to drive vaccine introduction in more countries in the immediate future. This should show even greater impact on reductions in diarrheal mortality over time.
